# Prevalence and determinants of physical frailty among people living in residential aged care facilities: a large-scale retrospective audit

**DOI:** 10.1186/s12877-022-03101-8

**Published:** 2022-05-14

**Authors:** Rachel Milte, Jasmine Petersen, Jo Boylan, Tim Henwood, Sarah Hunter, Belinda Lange, Michael Lawless, Stacey Torode, Lucy K. Lewis

**Affiliations:** 1 Caring Futures Institute, College of Nursing and Health Sciences, Flinders University, GPO Box 2100, Adelaide, SA 5001 Australia; 2 Southern Cross Care (SA, NT & Vic) Inc., PO Box 155, Glen Osmond, SA 5064 Australia

**Keywords:** older adults, frailty, aged care

## Abstract

**Background:**

Physical frailty is associated with increased risk of falls, hospitalisation and mortality. There is a dearth of information on physical frailty of older adults living in residential aged care. This study aimed to describe physical frailty in aged care residents and investigate possible determinants of frailty.

**Method:**

A retrospective audit of resident records was undertaken across 14 residential aged care facilities. Data were extracted on all consenting residents who had completed measures relating to frailty (Short Physical Performance Battery SPPB; grip strength). All data of the first record of measures were extracted, resident characteristics, and the time from admission to assessment. Summary statistics were completed. Differences between sub-groups were explored (Mann-Whitney U, Kruskall-Wallis Ranked tests). Associations between variables were explored with Chi-squared and Pearson correlations. Determinants of physical frailty were determined with linear regression analyses. Alpha (2-sided) was 0.05.

**Results:**

Data were extracted for 1241 residents (67% female), with a mean age of 86.0 (7.6) years. Males had a significantly lower time from admission to assessment of frailty (*p* ≤ 0.001). The average SPPB score was 4.1 (3.3), 75% of residents were frail and 19% pre-frail. Bivariate analyses indicated no significant relationships between grip strength and SPPB score, but significant differences for grip strength, where males were significantly stronger (males 20.2 ± 8.3 kg; females 12.4 ± 5.4 kg; *p* ≤ 0.001). There was a significant positive relationship between SPPB total score and grip strength, gender (p ≤ 0.001), and marital status (*p* = 0.049) and a negative relationship between time from admission to assessment and SPPB total score (*p* ≤ 0.001). There were significant negative relationships between gender (*p* ≤ 0.001) and age (*p* ≤ 0.001), and time from admission to assessment (*p* ≤ 0.001) with grip strength.

**Conclusion:**

Older adults living in residential aged care have a high level of physical frailty which may lead to increased risk of adverse outcomes. Time in the residential aged care setting and age appear to predict physical frailty. There is a need for a consistent battery of measures to continually monitor frailty and programs to address the high levels of frailty in residential aged care.

## Background

Population ageing is accelerating rapidly. Globally, in 2019, there were 703 million persons aged 65 years and older, with this number projected to increase exponentially, reaching 1.5 billion in 2050 [1]. As populations age, there is increased demand for aged care which encompasses a range of services, from home support, to long term care in Residential Aged Care Facilities (RACF). In Australia, more than 1.2 million people receive aged care services (approximately one quarter residing in RACF), and this is expected to grow two-fold by the middle of the century [2]. The aged care sector is expanding to match the increasing demands through the implementation of a ‘target provision ratio’, such that 125 aged care places will be provided per 1000 people aged 70 years or older in 2021–2, with most of these places in RACF [3].

People living in RACF are at a greater risk of physical frailty due to their age, functional impairment, and co-morbidities [4]. Frailty describes a condition where a person is at risk of adverse health outcomes and death [5], and can be physical or psychological, or a combination of both. There has been some effort to gain consensus on a definition of physical frailty, resulting in frailty being defined as: “*a medical syndrome with multiple causes and contributors that is characterised by diminished strength, endurance, and reduced physiologic function that increases an individual’s vulnerability for developing increased dependency and/or death*” [6]. Frailty is linked with adverse outcomes including falls, reduced functional independence, reduced quality of life, and premature mortality [7]. The dynamic nature of frailty provides opportunity for preventative and restorative interventions [8, 9], with frailty screening imperative to the implementation and timing of appropriate interventions [6]. Existing literature reports that a criterion commonly used to objectively screen frailty is the Fried frailty index which defines frailty as the presence of three or more of the following: shrinking, weakness, exhaustion, slowness, and low physical activity [10]. However, in practice, screening against these criteria can be impractical. There are many other tools that have been used to screen for frailty, including the Frailty in Nursing Homes (FRAIL-NH) scale [11], Rockwood Clinical Frailty Scale [12], Tilburg Frailty Indicator [13], Osteoporotic Fractures Frailty (SOF) Index [14] and the Short Physical Performance Battery (SPPB) [15]. Single markers of frailty have also been proposed, including physical markers such as grip strength [16] and physiological markers such as endocrine and inflammatory factors [17]. These screening tools and markers all have distinct and varying properties which can provide challenges in determining the overall prevalence of frailty in older adults. While some effort has been made to gain consensus on the use of simple screening tests to recognise and identify physical frailty or risk of frailty [6], there is currently no consensus on how, when and how often physical frailty should be assessed in RACF.

To date, existing research has largely reported on the prevalence of frailty in the community setting. A systematic review of 21 community-based studies reported that the weighted prevalence of frailty among 61,500 community-dwelling older persons (≥65 years) was 11% (range 4 to 59%) [18]. There is a paucity of literature investigating the prevalence of physical frailty in long term care settings such as nursing homes. A systematic review incorporating nine studies (total of 1373 residents aged ≥60 years) reported that the pooled estimate of frailty in long term care settings was 52% (range 19 to 76%) [4]. The included studies were conducted in Europe (*n* = 4), the Americas (*n* = 2), Asia (n = 2) and Africa (*n* = 1), with none from Oceania. One subsequent study has assessed the prevalence of frailty in RACF in Australia and reported that 61% of residents were frail [11]. Although this study provides some evidence for the prevalence of frailty in Australian RACF, modelling is yet to produce frailty estimates for RACF residents, limiting efforts to develop targeted health interventions for this population [19].

A small body of research has examined the determinants of frailty in community-dwelling older adults. For example, Gobbens et al., (2010) identified that older age, gender (being female), medium income, unhealthy lifestyle (e.g., low physical activity, poor eating habits) and multimorbidity predicted frailty [20]. Feng et al., (2017) documented that physical (e.g., Body Mass Index BMI, functional status) and psychological factors (e.g., depression, impaired cognition) also predict frailty among community-dwelling older adults [21]. A recent study has reported similar determinants of frailty (e.g., older age, being female, low income, unhealthy lifestyle, multimorbidity) in older adults admitted to hospital [22]. However, the determinants of frailty in older persons in RACF are currently unknown; an important limitation to the current literature given the prevalence of frailty in this population. Ascertaining the determinants of frailty among aged care residents is important to identify individuals most at risk, and therefore facilitate timely implementation of preventative and restorative interventions in this setting. Given the dearth of literature regarding prevalence and determinants of physical frailty in long term care settings for older adults, this study aimed to describe the level of physical frailty of older adults living in RACF in Australia and investigate possible determinants of frailty.

## Methods

This study used a retrospective audit design. Ethical approval was gained from the Flinders University Human Research Ethics Committee (protocol no. 2476).

### Participants

An audit of the existing electronic records of residents in 14 RACF in metropolitan Adelaide, South Australia was completed in 2020. Relevant data were extracted from the records of residents who had consented to an organisational agreement on admission to residential care, allowing data collected as part of routine care to be de-identified and shared for research purposes. Facility locations were ranked based on deciles according to the Socio-Economic Indexes for Australia (SEIFA) Index of Relative Socio-Economic Disadvantage for postal areas. A lower decile (e.g. 1) indicates greater socio-economic disadvantage for an area, while a higher decile (e.g. 10) indicates the lowest level of disadvantage [23].

### Procedure

Data were extracted from all consenting residents who had completed measures of frailty and had these recorded on their electronic record over the previous 5 years (2016–2020). At the aged care organisation, all residents underwent standardised measures prior to development of a tailored exercise program. For the purposes of the audit, all available electronic data of the first occasion of measures relating to frailty since admission for each resident were extracted, de-identified and forwarded to the research team for analysis.

### Measures

Resident characteristics were extracted including admission date, gender, marital status, pension status, date of birth, and use of a walking aid. The time from admission to assessment of frailty was recorded. Two outcomes relating to physical frailty were routinely collected and recorded across the participating facilities, the SPPB and grip strength, and therefore included in the audit.

#### Short Physical Performance Battery

The SPPB is a valid and reliable measure of lower extremity function [15] and frailty in older adults, demonstrating fair to moderate agreement with Fried’s Frailty Phenotype [24]. Staff at the sites followed a set protocol for administering the SPPB. The SPPB measures balance, gait speed and strength and is scored out of a maximum of 12, with a maximum of four points for each of the components. Balance was assessed progressively with the instructor first describing and demonstrating each of the three stages (feet side by side, semi-tandem stance and tandem stance), with the resident then provided with the opportunity to complete the movement. Residents only progressed to the next stage if they successfully completed the prior stage for 10 seconds. Gait speed was measured for all residents able to mobilise. Residents were asked to walk four metres independently (+/− walking aid) with the instructor timing. The best of two trials was recorded. Strength was measured firstly with a single chair stand. Residents who could complete this safely progressed to assessment of a repeated chair stand. Residents were asked to stand up from the chair as quickly as possible five times without stopping or using their arms (arms crossed across the chest). The time to complete five stands was recorded. Participants were classified as frail (SPPB 0 to 6); pre-frail (SPPB 7 to 9) and non-frail (SPPB 10 to 12) [25].

#### Grip strength

All residents underwent bilateral assessment of grip strength as a marker of physical frailty [16]. Grip strength is useful for approximating overall muscle strength in older adults and has been shown to be a predictor of functional limitation [26], Activities of Daily Living (ADL) dependence [27] and all-cause mortality [28]. The test was completed with residents seated on a chair with armrests [29]. Residents were instructed to sit with their hips and knees as close to 90 degrees as possible, with the testing forearm supported by the armrest. A dynamometer was used, with the instructor resting the base of the device on their hand for support to reduce the impact of gravity on peak strength. The resident was asked to squeeze the dynamometer with maximum effort for three seconds. No other body movement was allowed, and encouragement was provided. Three trials each side were completed with at least 30 seconds rest in-between. The best of the trials on each side was recorded. The highest grip strength (from either side) was included in the analysis.

### Data management and analysis

Data were extracted into Microsoft Excel and exported into IBM SPSS Statistics version 27 for analyses. Descriptive and summary statistics were calculated and presented for the socio-demographic variables for the total sample and for subgroups according to gender. Given the continuous variables were found to be non-normally distributed based on the Shapiro-Wilk test, the Mann-Whitney U test or the Kruskall-Wallis Ranked test were used to test for differences in variables between subgroups. Chi-squared was used to test for associations between categorical variables. Both Spearman and Pearson correlations were conducted to test for relationships between continuous variables. The size of correlation coefficients was interpreted as negligible (0.00 to 0.30), low (> 0.30 to 0.50), moderate (> 0.50 to 0.70) and high (> 0.70 to 0.90). Linear regression analysis was conducted to investigate which covariates influenced the dependent variables of SPPB total score and grip strength. Grip strength was dichotomised according to the European Working Group on Sarcopenia in Older People (EWGSOP-II) criteria to describe the prevalence of sarcopenia in the sample [30], then retained as a continuous variable in the analysis, with gender as a covariate to account for the well-known gender difference in hand grip. Given the potential for clustering in the data based on the RACF sites the participants were residing in at the time of data collection, we also investigated the need for a multi-level regression model which could account for any variation between the sites. Initial analysis indicated that there was no significant variation in the intercepts for the model based on the sites, and therefore we returned to the standard linear regression model. The models included covariates that were associated with the grip strength or SPPB score at a significance level of ≤0.10. Residual versus predicted value scatterplots were inspected to check for any violation of the assumptions of linearity and homoscedasticity, while the assumption of normality of the residuals was assessed through inspection of the normal probability plots of the regression standardised residuals. We examined the standardised residuals for any cases with values greater than 3 or − 3, and for any Cook’s Distance values greater than 1 to identify any outliers having undue influence on the model. A 2-sided alpha of ≤0.05 was considered statistically significant.

## Results

### Participant characteristics

Data were extracted for 1241 residents (67% female) who were subsequently included in the analysis (Table 1). Overall, residents were aged 86.0 years (SD 7.6), with males significantly younger than females (*p* ≤ 0.001). Males had a significantly lower wait time from admission to their first assessment of physical frailty (*p* ≤ 0.001), and were more likely to be single, widowed or separated (*p* ≤ 0.001). Most residents were single (68%), on a full pension (69%) and mobilised with a walking aid (73%).

**Table 1:** Participant characteristics for the whole sample and by gender

Characteristics	All *n* = 1241	Males *n* = 407	Females *n* = 834	Test of difference
Age at assessment (years)^a^ mean (SD)	86.0 (7.6)	84.8 (8.1)	86.6 (7.3)	−3.3, **0.0008**
Time from admission to assessment (months)^a^ mean (SD)	19.7 (31.4)	13.7 (23.1)	22.6 (34.5)	−4.2, **0.000**
Marital status n (%)				
Single/widowed/separated	603 (67.8)	150.0 (51.2)	453 (75.9)	−7.4, **0.000**
Married/relationship^a^	287 (32.2)	143.0 (48.8)	144 (24.1)	
Pension status^b^ n (%)				
None	211 (17.0)	72.0 (17.7)	139 (16.7)	
Part	175 (14.1)	71.0 (17.4)	109 (12.5)	6.4 (2), **0.041**
Full	855 (68.9)	264.0 (64.9)	591 (70.9)	
Uses a walking aid^a^ n (%)	456 (73.2)	135 (68.2)	321 (75.5)	1.9, 0.054

^a^ Denotes a non-parametric two-sample Mann-Whitney U test with the results reported as: z, *p*-value; ^b^Denotes a Chi-square test with the results reported as: Chi-square (df), *p*-value; Bold represents significance at *p* ≤ 0.05

### Description of physical frailty

Table 2 summarises the measures of physical frailty for all residents and by gender. The highest proportion of residents were unable to achieve the first stage of balance testing (29%), walk four metres (27%) or stand repeatedly from a chair (65%). The average time to walk four metres was 12.0 seconds (SD 45.5). The average SPPB score for all residents was 4.1 (SD 3.3) out of 12, with 75% of residents identified as frail, 19% pre-frail and 7% non-frail. There were no significant differences by gender in markers of physical frailty, except for grip strength, where males were significantly stronger than females (*p* ≤ 0.001). Seventy-four per cent of all residents were classified as sarcopenic (EWGSOP-II) [30].

**Table 2:** Description of physical frailty for the whole sample and by gender

Physical frailty measure	All *n* = 1241	Males *n* = 407	Females *n* = 834	Test of difference
Balance^a^ n (%)				
Unable	358 (28.8)	115 (25.3)	243 (29.1)	3.8 (4), 0.440
Side-by-side 10 seconds	165 (13.3)	48 (11.8)	117 (14.0)	
Semi-tandem stand 10 seconds	268 (21.6)	84 (20.6)	184 (22.1)	
Tandem stand 3–9.99 seconds	187 (15.1)	71 (17.4)	116 (13.9)	
Tandem stand 10 seconds	263 (21.2)	89 (21.9)	174 (20.9)	
Gait speed (4 m)^a^ n (%)				
Unable	338 (27.2)	112 (27.5)	226 (27.1)	5.4 (4), 0.245
≥ 8.71 seconds	303 (24.4)	88 (21.6)	215 (25.8)	
6.21 to 8.70 seconds	266 (21.4)	87 (21.4)	179 (21.5)	
4.82 to 6.20 seconds	156 (14.3)	70 (17.2)	108 (13.0)	
≤ 4.81 seconds	156 (12.6)	50 (12.3)	106 (12.7)	
Gait speed (seconds)^a^ mean (SD)	12.0 (45.5)	12.3 (44.7)	11.8 (46.0)	−1.4, 0.1667
Strength (repeated chair stand)^a^ n (%)				
Unable or not completed in 60 seconds	800 (64.5)	260 (63.9)	540 (64.8)	1.8 (4), 0.767
16.70 to 50.00 seconds	250 (20.1)	82 (20.2)	168 (20.1)	
13.70 to 16.69 seconds	74 (6.0)	29 (7.1)	45 (5.4)	
11.20 to 13.69 seconds	58 (4.7)	19 (4.7)	39 (4.7)	
≤ 11.19 seconds	59 (4.8)	17 (4.2)	42 (5.0)	
SPPB Total Score^a^ mean (SD)	4.1 (3.3)	4.2 (3.3)	4.1 (3.9)	0.8, 0.4037
Non-frail 10 to 12 n (%)	86 (6.9)	31 (7.6)	55 (6.6)	
Pre-frail 7 to 9 n (%)	229 (18.5)	78 (19.2)	151 (18.1)	
Frail 0 to 6 n (%)	926 (74.6)	298 (73.2)	628 (75.3)	
Grip strength, strongest (kg)^a^ mean (SD)	14.9 (7.5)	20.2 (8.3)	12.4 (5.4)	16.5, **0.0000**
Sarcopenic (EWGSOP-II criteria) n (%)	916 (74)	599 (72)	317 (78)	

EWGSOP European Working Group on Sarcopenia in Older People; ^a^ Denotes a non-parametric two-sample Mann-Whitney U test with the results reported as: z, *p*-value; ^a^Denotes a Chi-square test with the results reported as: Chi-square (df), *p*-value; Bold represents significance at *p* ≤ 0.05

### Determinants of physical frailty

Table 3 provides the associations between the SPPB total score and grip strength and pension and marital status of the residents. We identified a significantly higher grip strength in those who were married (16 kg, IQR 11–21) compared to those who were single, widowed or separated (13 kg, IQR 10–18, *p* ≤ 0.001).

**Table 3:** Associations between physical frailty measures and independent categorical variables

Independent variable	SPPB total score Median (IQR)	Kruskall-Wallis Rank Test Adjusted Chi-squared (***p***-value)*	Grip Strength Median (IQR)	Kruskall-Wallis Rank Test Adjusted Chi-squared (***p***-value)*
Pension status				
None	4 (2 to 7)	2.517 (0.2840)	14 (11 to 20)	1.839 (0.3988)
Part	4 (0 to 7)		15 (10 to 20)	
Full	4 (1 to 6)		14 (10 to 19)	
Marital status				
Single/widowed/separated	4 (0 to 6)	2.236 (0.1325)	13 (10 to 18)	19.49 (**0.0001**)
Married/relationship	4 (1 to 7)		16 (11 to 21)	

*The test of difference in Table 3 uses a Kruskal-Wallis rank test and reports the adjusted Chi-squared statistic (adjusted for ties), and the *p*-value; Bold represents significance at *p* ≤ 0.05

Table 4 presents the Pearson correlations between the physical frailty measures and independent variables. For the SPPB total score, there was a statistically significant low correlation with grip strength (*p* ≤ 0.01), a negligible correlation with time from admission to assessment (*p* ≤ 0.01), and age at assessment (*p* < 0.05). There were statistically significant negligible correlations between grip strength and time from admission to assessment (*p* ≤ 0.01), and age (*p* ≤ 0.01). Time from admission to assessment was negligibly associated with age (*p* ≤ 0.01), and age was negligibly associated with SEIFA ranking of location (*p* < 0.05). Correlations undertaken using Spearman Rho gave similar results and are therefore not included in the manuscript.

**Table 4:** Pearson correlations between physical frailty measures and independent continuous variables

	SPPB total score	Grip strength (strongest)	Time from admission to assessment (months)	Age at assessment (years)	SEIFA ranking of location
SPPB total score		0.351***	−0.165***	−0.066**	−0.013
Grip Strength (strongest)			− 0.193***	−0.241***	− 0.037*
Time from admission to assessment (months)				.130***	0.068*
Age at assessment (years)					0.081**

*** *p* ≤ 0.01; **p < 0.05, *p < 0.10

Table 5 presents the results of the linear regression analysis with SPPB total score as the dependent variable. There was a significant positive relationship between grip strength and SPPB total score (*p* **≤** 0.001) and a negative relationship between time from admission to assessment and SPPB total score (*p* **≤** 0.001). There was also a significant relationship identified between gender (with females having a higher SPPB total score, *p* ≤ 0.001) and a relationship between marital status and SPPB score (with those who were single having a higher SPPB total score, *p* = 0.011) when all other factors in the model were held constant. We did not identify significant independent relationships between age at assessment or SEIFA ranking of service location and SPPB total score. Table 6 presents the results of the linear regression analysis for grip strength as the dependent variable. There were significant negative relationships between gender (*p* **≤** 0.001) and age at assessment (*p* **≤** 0.001), and time from admission to assessment (*p* = 0.049) with grip strength. There was also a significant positive relationship between SPPB total score and grip strength (*p* **≤** 0.001).

**Table 5:** Linear regression analyses predicting SPPB total score among people living in residential aged care

Variable	Standardised Coefficient	*p*-value	95% CI (Lower)	95% CI (Higher)
Grip strength (Strongest)	0.437	**≤0.001**	0.160	0.223
Time from admission to assessment (months)	−0.121	**≤0.001**	−0.019	−0.006
Gender	0.184	**≤0.001**	0.792	1.775
Marital status	0.082	**0.011**	0.134	1.012
Age at assessment (years)	0.026	0.418	−0.016	0.038
SEIFA rank of location	0.007	0.812	−0.078	0.099
N	890			
R	0.171			
Adjusted R squared	0.165			

Reference Category for Gender = Males and Marital status = married, Bold indicates significance at *p* ≤ 0.05

**Table 6:** Linear regression analyses predicting grip strength among people living in residential aged care

Variable	Standardised Coefficient	*p*-value	95% CI (Lower)	95% CI (Higher)
SPPB total score	0.323	**≤0.001**	0.616	0.856
Gender	−0.450	**≤0.001**	−8.020	−6.310
Marital status	−0.033	0.236	−1.383	0.342
Age at assessment (years)	−0.160	**≤0.001**	−0.208	−0.105
Time to admission assessment (months)	−0.054	0.049	−0.026	0.000
SEIFA rank of location	−0.012	0.652	−0.214	0.134
N	890			
R	0.389			
Adjusted R squared	0.384			

Reference Category for Gender = Males and Marital status = married, Bold indicates significance at *p* ≤ 0.05

## Discussion

The aim of this study was to describe the level of, and determinants of physical frailty of older adults living in RACF. A large-scale retrospective audit was completed with data extracted regarding resident characteristics, together with existing outcomes relating to physical frailty. Most residents were single, female, on a full pension and walked with a walking aid. There was a very high prevalence of physical frailty in this sample, with increased time from admission to assessment, gender (male) and marital status (married) predicting lower SPPB scores; and gender (female), increased age and time from admission to assessment predictive of reduced grip strength.

This study represents one of few published examples examining physical frailty in a large sample of older adults living in RACF. While it has long been assumed that older adults admitted to long term care are physically frail, the extent and characteristics of the level of frailty have not been as extensively evaluated as other sectors, for example among people admitted to hospital or older adults living in the community. In our study, high levels of physical frailty were widespread in the sample, across all included measures. The average SPPB score for all residents was 4.1 out of 12 (SD 3.3), with over half of all residents (56%) scoring a four or below and almost all residents (97%) scoring 10 or under. Previous research has suggested that a total score of 10 or under in the SPPB is predictive of mobility disability [31] and all-cause mortality [32] in community dwelling older adults. In addition, older adults who score between four and six have over four times the risk of developing ADL disability within 4 years in comparison to those who score between 10 or above [33]. The residents in our sample are at very high risk of all-cause mortality, ADL and mobility disability according to their scores for this measure of physical frailty. Poor performance on the SPPB has also been reported in a 2018 study of Australian older adults living in RACF, with a mean score of 3.5 (SD 2.4) [34]. A further study of Swiss older adults admitted to post-acute care in nursing homes reported a slightly higher, albeit low, mean SPPB score of 5.2 (SD 2.9), but this may be because these older adults were specifically selected for entry into a program aiming to improve their physical function, rather than drawn from the general nursing home population [35].

Grip strength is routinely used as a simple, inexpensive clinical marker of wellbeing in older adults [29]. Reduced grip strength has been shown to be predictive of hospital length of stay, higher risk of institutionalisation, functional limitation, and mortality in older adults [26–28, 36]. A systematic review of grip strength cut-off values in older adults (≥65 years) reported that most studies including non-Asian participants used cut-off values for low grip strength of 30 kg for men and 20 kg for women [37]. The average grip strength for both male and female residents in this sample was therefore low, indicating an increased risk of functional limitation and death. This finding is consistent with previous studies, indicating a lower grip strength in older adults residing in nursing homes compared with those in hospital or in the community [38], and nursing home residents in Spain (10.23 (SD 6.49) kg) and in Australia (16.5 (SD 7.7) kg) [34, 39].

A high proportion of our sample (73%) used a walking aid, and a large proportion were unable to walk four metres (27%). Reduced gait speed was also evident in our sample, with a mean gait speed of 12 seconds over four metres, and a large proportion of the sample either unable to complete the task or taking more than 6.2 seconds (73%). The relationships between reduced mobility and poor gait speed and outcomes such as mortality, morbidity and increased health care utilisation have been well demonstrated [40–43]. Similarly other studies have identified high levels of poor mobility among nursing home residents. Bravo-Jose et al. (2018) found 65% of their sample of Spanish older adults living in nursing homes were either confined to a wheel-chair or walking using a walking aid [39]. Overall, the findings of this study add to the growing evidence of the pervasiveness and extent of physical frailty in the residential aged care setting both in Australia and other high-income nations with relatively well-developed health and aged care systems.

We identified statistically significant relationships between the SPPB score and grip strength, and time from admission to assessment in the bivariate analysis. Relationships between SPPB score and grip strength and time from admission remained in the linear regression model when gender, marital status, aged and assessment and SEIFA rank of location were included in the model. Relationships between SPPB score and gender (with females having a higher SPPB score than males) and marital status (with those who were single having a higher SPPB score than those who were married) were identified when all other factors in the model were held constant. This could be due to the strong relationship identified between gender and grip strength, and marital status and grip strength, masking relationships between these factors and the SPPB score in the bivariate analysis. We also identified significant relationships between grip strength and time from admission, age at assessment, marital status, and gender in the bivariate analysis. Relationships between grip strength and SPPB score, age at assessment, gender and time from admission to assessment remained in the linear regression analysis, but the relationship with marital status was insignificant. This could be due to the significant relationship between gender and marital status, with females significantly more likely to be single, and the relationship becoming insignificant once gender was accounted for in the model.

Previous studies among older adults have indicated relationships between SPPB scores and level of physical and sedentary activity, barriers to good nutritional intake, knee-extensor muscle strength, handgrip strength, appendicular lean mass, inflammatory markers, and vitamin D levels [44–48]. Interestingly, relationships between grip strength and SPPB have been shown to become insignificant after adjustment for factors such as age, gender, BMI, cognitive impairment and nutritional status [49]. In addition, being overweight or obese does not preclude older adults from poor physical function, with relationships between higher BMI and increased body fat percentage positively associated with SPPB scores [47]. Most previous studies have demonstrated these relationships in community dwelling older adults, with or without chronic conditions. Our study has demonstrated relationships between markers of physical function, age, and gender among older adults living in RACF, a group who are likely to be significantly frailer and in poorer health overall than community-dwelling older adults. A key finding of our analysis has been the negative relationship between time from admission to assessment of physical frailty and both SPPB score and grip strength. This could indicate that over time in RACF, older adults’ physical function declines. A previous systematic review identified physical rehabilitation had positive but relatively small effects on physical function, mobility, and walking speed among older adults in long-term aged care, and possible effects on strength, flexibility, balance and mood [50, 51]. However, whether these effects are maintained over the long term, applicable to all residents, or whether the potential effectiveness of physical rehabilitation interventions is reduced at higher levels of decline is currently unknown. There remains a need for more research exploring interventions to prevent and treat physical frailty among older adults living in RACF, including identifying interventions which best suit different groups of residents according to clinical or cognitive status, and optimal timing of interventions.

Correlation analyses identified a relationship between grip strength and SPPB total score, which was further supported by the results of the regression. However, the size of the association was low, indicating that while these two markers of physical frailty are related, there is not complete overlap between the two. While grip strength and SPPB score are related, they provided independent information about the physical frailty in this sample of older adults in RACF. There is currently no consensus regarding core outcomes for assessing physical frailty among older adults in this setting, and this study indicates that a range of measures may be required to provide a clear and accurate picture. Given the pervasiveness and extent of physical frailty in this setting and the associated risks, there is a clear need for comprehensive, routine, and regular assessment of frailty to ensure declines do not go undetected and untreated.

This study had several strengths. The retrospective audit included a large sample size of over 1200 residents across multiple sites. Measures of physical frailty were assessed at each site using a standardised protocol, with valid and reliable measures. There were also some limitations. These data were extracted from one aged care provider, in one Australian state, meaning that the results may not be generalisable to other providers or regions. There was wide variability in the length of time between admission and assessment of physical frailty, and a lack of data on other co-morbidities which may have impacted the findings. It could be postulated that, given the mean age of participants in this study, the majority of residents would likely have five or more co-morbid conditions, when compared with other populations of similar age and setting [52], with the most common conditions being hypertension, depression, anaemia, cardiovascular disease and dementia. Finally, the outcomes relating to physical frailty used in this study were limited to those that were routinely assessed and recorded for all residents across the participating facilities. The SPPB is a commonly used measure in the residential aged care setting. The SPPB has been shown to be a valid and reliable measure of physical frailty in older adults, demonstrating fair to moderate agreement with Fried’s Frailty Phenotype [24], predictive ability comparable to the Frailty Phenotype and Frailty Index [53], ability to identify physical frailty and predict geriatric syndromes [54], and screen frailty in older adults comparable to the Fried Frailty Phenotype [55]. It is recognised that there are a number of other commonly used and validated frailty tools that could be applied in this setting [6, 11]. There is a clear need for consensus regarding the timing and methods of frailty screening applied in residential care, with consistent methods potentially leading to the establishment of normative data with consideration of clinical and functional significance in this setting.

## Conclusion

The findings from this study indicated that there was a high level of physical frailty and pre-frailty among older adults living in residential aged care prior to undertaking any intervention, indicating an increased risk of mortality and disability. There is a clear need for routine and repeated assessment of physical frailty in this setting, as well as programs to address the high levels of frailty and pre-frailty.

## Data Availability

The datasets used and/or analysed during the current study are available from the corresponding author on reasonable request.

## Abbreviations

ADL: Activities of Daily Living
BMI: Body Mass Index
IQR: Inter-Quartile Range
RACF: Residential Aged Care Facilities
SD: Standard Deviation
SEIFA: Socio-Economic Indexes for Australia
SPPB: Short Physical Performance Battery
